# “Think Neurodevelopmental”: Why Missing BIF/ID/Autism Before Therapy Risks Symptom-Focused Mismanagement in Youth Mental Health

**DOI:** 10.1192/j.eurpsy.2026.10567

**Published:** 2026-07-31

**Authors:** L. López Gómez-Miguel, A. Gutiérrez Fernández de Velasco, Á. Privado Aranda, P. Bang Carrascosa, J. García Iglesias, M. González San José, M. Rodriguez Fernández, M. I. Montañés Rodríguez, A. Ladera de la Oliva, A. S. Benito Martin-Preciados, L. M. F. Oliva, M. Andrade Arango, E. García Martín de la Fuente, R. C. Ramos, M. D. R. D. F. Rodríguez, L. P. Ayuso Morales

**Affiliations:** 1 Hospital Universitario de Toledo, Toledo, Spain; 2 Programa de Transición y Primeros Consumos; 3 Programa de Transición a la vida adulta y Primeros consumos, Hospital Universitario de Toledo, Toledo, Spain; 4 Clínica Nuestra Señora de La Paz, Bogotá, Colombia

## Abstract

**Introduction:**

An increasing number of adolescents present to mental health services with anxiety, depression, or self-harming behaviors. In a significant subset, these symptoms conceal undiagnosed neurodevelopmental conditions—such as borderline intellectual functioning (BIF), mild intellectual disability (ID), or autism spectrum disorder (ASD)—that were not identified during childhood. Many of these youths advanced academically and socially despite substantial effort, which delayed recognition by pediatricians and schools. Without acknowledging these hidden developmental profiles, clinicians risk applying symptom-focused therapies that are ill-suited to the adolescent’s cognitive and adaptive capacities.

**Objectives:**

(1) To highlight the impact of missed childhood diagnosis of BIF, mild ID, or ASD in adolescents presenting with emotional or behavioral symptoms. (2) To demonstrate how undetected neurodevelopmental conditions undermine standard psychotherapeutic approaches. (3)To propose systematic neurodevelopmental screening prior to intervention initiation in adolescent mental health care.

**Methods:**

We conducted a narrative review (2023–2025) of recent meta-analyses on adapted psychotherapy in ASD and ID, conceptual reviews of BIF, and major international guidelines (NICE, NHS England). We analyzed recommendations on neurodevelopmental screening and psychotherapeutic adaptations for adolescents.

**Results:**

1. **Missed in childhood:** Many adolescents with low IQ or autistic traits escaped earlier detection, progressing academically at the cost of hidden distress.
2. **Secondary presentations:** In adolescence, they present with anxiety, depression, or self-harm, symptoms that overshadow the underlying condition.
3. **Therapy works—when adapted:** Evidence supports the efficacy of adapted psychotherapies (simplified CBT, visual supports, structured pacing, family involvement) when neurodevelopmental conditions are recognized.
4. **Guideline gap:** Although guidelines recommend adaptations, they rarely require clinicians to systematically rule out neurodevelopmental conditions prior to starting standard therapy.

**Conclusions:**

The failure to detect neurodevelopmental disorders early partly explains why many adolescents show poor response to conventional, symptom-focused treatments. Unless mental health services incorporate routine cognitive and adaptive screening before pychotherapy or pharmacotherapy, treatments risk addressing symptoms while ignoring root causes. This raises a necessary debate: should adolescent mental health care continue to apply one-size-fits-all protocols, or move toward precision psychotherapy tailored to each individual’s neurodevelopmental profile?

**Disclosure of Interest:**

None Declared

